# Interaction Analysis Based on Shapley Values and Extreme Gradient Boosting: A Realistic Simulation and Application to a Large Epidemiological Prospective Study

**DOI:** 10.3389/fnut.2022.871768

**Published:** 2022-07-18

**Authors:** Nicola Orsini, Alex Moore, Alicja Wolk

**Affiliations:** ^1^Department of Global Public Health, Karolinska Institutet, Stockholm, Sweden; ^2^Managed Self Ltd T/A Klarity, Bournemouth, United Kingdom; ^3^Institute of Environmental Medicine, Karolinska Institutet, Stockholm, Sweden; ^4^Department of Surgical Sciences, Orthopaedics, Uppsala University, Uppsala, Sweden

**Keywords:** extreme gradient boosting, Shapley values, interaction, simulation study, prospective cohort design

## Abstract

**Background:**

SHapley Additive exPlanations (SHAP) based on tree-based machine learning methods have been proposed to interpret interactions between exposures in observational studies, but their performance in realistic simulations is seldom evaluated.

**Methods:**

Data from population-based cohorts in Sweden of 47,770 men and women with complete baseline information on diet and lifestyles were used to inform a realistic simulation in 3 scenarios of small (OR_M_ = 0.75 vs. OR_W_ = 0.70), moderate (OR_M_ = 0.75 vs. OR_W_ = 0.65), and large (OR_M_ = 0.75 vs. OR_W_ = 0.60) discrepancies in the adjusted mortality odds ratios conferred by a healthy diet among men and among women. Estimates were obtained with logistic regression (L-OR_M;_ L-OR_W_) and derived from SHAP values (S-OR_M;_ S-OR_W_).

**Results:**

The sensitivities of detecting small, moderate, and large discrepancies were 28, 83, and 100%, respectively. The sensitivities of a positive sign (L-OR_W_ > L-OR_M_) in the 3 scenarios were 93, 100, and 100%, respectively. Similarly, the sensitivities of a positive discrepancy based on SHAP values (S-OR_W_ > S-OR_M_) were 86, 99, and 100%, respectively.

**Conclusions:**

In a realistic simulation study, the ability of the SHAP values to detect an interaction effect was proportional to its magnitude. In contrast, the ability to identify the sign or direction of such interaction effect was very high in all the simulated scenarios.

## Introduction

SHapley Additive exPlanations (SHAP) values have been recently proposed to facilitate the explanation of results obtained from supervised machine learning algorithms (1). Interaction between the predictors of an outcome is often of interest in epidemiological and public health research. For example, in nutritional epidemiology, the association of a dietary factor with the future occurrence of a particular disease may vary according to another factor. However, this is not a secondary analysis. It is the main research question of substantial interest.

An appealing feature of SHAP methods is that an assessment of interaction can be based on visualizations rather than complex numerical derivations (2). This facilitates a graphical illustration of how the association between one exposure and the outcome may vary along the distribution of another factor. SHAP values are computed on an individual level to explain the importance of the predictors (3). In epidemiological research, the possibility to utilize SHAP values to derive a concise numerical summary at the population-level, which is also capable of expressing the direction and magnitude of interaction effects, would be helpful for interpreting results obtained from machine learning methods.

The field of nutritional epidemiology—characterized by a lack of randomly assigned exposures, relatively modest associations, and possibly complex dependencies between genetic, lifestyle, environmental, and socio-demographic factors—can represent an ideal setting to evaluate the insights provided by SHAP methods derived from popular tree-based machine learning algorithms. If health-related decisions or public recommendations are going to be based on applications of these methods, then it is important to understand their performance in controlled, yet realistic, scenarios. It is important to evaluate the ability of SHAP methods to pinpoint a specific aspect of the data generating mechanism that underlies the observed outcomes, that is, a genuine variation of an exposure-outcome association across levels of another factor.

Data from a large population-based Swedish Mammography Cohort and a Cohort of Swedish Men were used to inform a realistic Monte-Carlo simulation focusing on interaction effects. To complement standard SHAP-based visualizations of dependencies between predictors, we derived a summary measure of exposure effect from SHAP values to facilitate comparisons with conditional odds ratios estimated in multivariable logistic regression models. This simulation study was used to evaluate the ability of SHAP methods to correctly indicate an interaction between healthy diet and female sex when predicting all-cause mortality.

## Materials and Methods

### Study Population

This study included participants from two large population-based cohorts of Swedish men and women, the Cohort of Swedish Men (COSM) and the Swedish Mammography Cohort (SMC) (4). Briefly, a total of 48,850 men and 39,227 women responded to the 1997 questionnaires and were included in this study. We excluded participants with diabetes, cancer, or cardiovascular disease at baseline. Furthermore, participants with any missing data on healthy diet, sleep duration, daily walking, alcohol consumption, smoking, cohabitation, body mass index, waist circumference, and educational level were automatically excluded from the analysis. The analytical sample was based on 47,770 participants (23,045 women and 24,725 men), aged 45–83 years. Descriptive statistics of the participants are presented in the Supplementary Material.

### Predictors of All-Cause Mortality

Diet was assessed using a 96-item food frequency questionnaire. Quality of diet was assessed by recommended food score based on 36 items and non-recommended food score based on 16 items (5). A binary indicator for a healthy diet was obtained by combining recommended food items (top quartiles) and non-recommended food items (bottom quartiles); otherwise not healthy. Age (<65; 65+ years), sex (woman; man), sleep duration (7 h; either <7 or >7 h), daily walking (never or <20 min/day; >20 min/day), smoking status (never; former or current), moderate total alcohol (including wine, beer, and spirits) intake (5–10 g/day for women and 5–20 g/day for men; either below or above such intervals), living with someone (yes; no), body mass index (≥20; <20 kg/m^2^), small waist circumference (<88 cm for women and <102 cm for men), and educational level (high school/university; primary) were also assessed at baseline with a self-administered questionnaire.

### Case Ascertainment and Follow-Up

Data on death was collected through linkage of the COSM and SMC data to the Swedish Cause of Death Register at the National Board of Health and Welfare (6). Over 20 years of follow-up, from January 1, 1998 to December 31, 2017, 21,978 deaths (9,566 in women and 12,412 in men) were documented in the analytical sample size of 47,770 participants.

### Monte-Carlo Simulation

The characteristics of the COSM and SMC data were used to inform the parameters underlying a Monte-Carlo simulation of a prospective cohort study. Descriptive statistics are provided in the Supplementary Material.

The interaction mechanism of interest is that the association of a healthy diet with decreased mortality, as measured by the odds ratio, is stronger among women (denoted as OR_W_) than men (denoted OR_M_), while accounting for possible differences with respect to age, body mass index, waist circumference, physical activity, smoking, alcohol consumption, education, cohabitation, and sleeping time. Given a fixed sample size of 47,770 persons, we considered 3 scenarios: small (OR_M_ = 0.75 vs. OR_W_ = 0.70), moderate (OR_M_ = 0.75 vs. OR_W_ = 0.65), and large (OR_M_ = 0.75 vs. OR_W_ = 0.60) discrepancy by sex in the adjusted inverse association of healthy diet with mortality risk.

To summarize the estimates obtained in 1,000 replications under the 3 scenarios of a genuine interaction effect, the first quantity of interest was the fraction of sample realizations that are correctly indicated as incompatible with the hypothesis of no interaction effect. This is the simulated sensitivity for a certain discrepancy (also known as statistical power). Ignoring the precise magnitude of the discrepancy and focusing only on its sign, the second quantity of interest was the fraction of studies in which the estimated mortality adjusted odds ratio conferred by a healthy diet is correctly estimated to be greater among women than men. This is the simulated sensitivity of a positive discrepancy.

### Data Analysis

The association between healthy diet (yes/no) and mortality risk according to sex (men/women) while adjusting for other important predictors was estimated using a traditional logistic regression model and by SHAP values based on extreme gradient boosting.

Extreme Gradient Boost (XGBoost) is a powerful supervised learning method that is well suited to tabular datasets (7). XGBoost chains together decision trees, with each tree trained to predict the previous tree's residuals, commonly known as gradient boosting. There are several hyperparameters controlling XGBoost. To maximize the accuracy of XGBoost these hyperparameters must be optimized. In this study the following hyperparameters were optimized before training our XGBoost model: the number of estimators (decision trees), the maximum depth of a given decision tree, the minimum child weight in a decision tree. The objective logistic link function was specified in the XGBoost classifier.

Shapley values originated as a concept in 1953 from cooperative game theory (8). Early surveys by Tijs et al. (9), Roth (10), and Winter (11) offer a review of the large number of studies that has grown out from the Shapley's seminal paper. Recently, Algaba et al. (12) and the references therein provide a volume devoted to the modern development and applications of the Shapley value in game theory and operations research, decision-making, and applied socio-economics research in various fields (13). In line with this growing literature, Lundberg et al. (1), Molnar (14), and Molnar et al. (15) propose applying the Shapley value in machine learning.

SHAP values facilitate the explanation of highly non-linear models, such as XGBoost, breaking down the impact of input features on prediction (1, 3, 14). SHAP values can be calculated by observing the change in a model's output when each feature is added sequentially. By considering all possible combinations of features, this approach ensures that complex interactions between inputs are captured (3). These interactions explain why two individuals with identical feature values may have different SHAP values associated with those features.

### Adjusted Odds Ratios Based on Logistic Regression

The adjusted mortality odds ratio conferred by a healthy diet among men (L-OR_M_) was obtained by taking the exponential value of the regression coefficient of healthy diet in a logistic regression model. An estimate of the adjusted mortality odds ratio conferred by healthy diet among women (L-OR_W_) was obtained by taking the exponential value of the estimated regression coefficient of healthy diet, plus the estimated regression coefficient of the product term between healthy diet and female sex. A two-sided Wald-type statistical test for the hypothesis of no interaction effect—that is, a regression coefficient of the interaction term equal to zero—was conducted with reference to a standard normal distribution. The result of this statistical test, as a measure of compatibility between data and hypothesis, was used to evaluate the sensitivity of the certain discrepancies previously described.

### Adjusted Odds Ratios Based on SHAP Values

Since individual SHAP values are represented as changes in the unit of log-odds, relative to an expected referent (16), a summary of such values may complement graphical illustrations based on dependence plots. The average of the individual SHAP values was first computed for each of the four possible combinations of healthy diet and sex. The SHAP-based adjusted mortality odds ratio comparing healthy diet vs. not-healthy diet was defined as the exponential value of the difference between the average SHAP values of healthy diet and the average SHAP values of not a healthy diet among men (S-OR_M_) and women (S-OR_W_), respectively.

## Results

### Simulation Study

**Figure 1:**
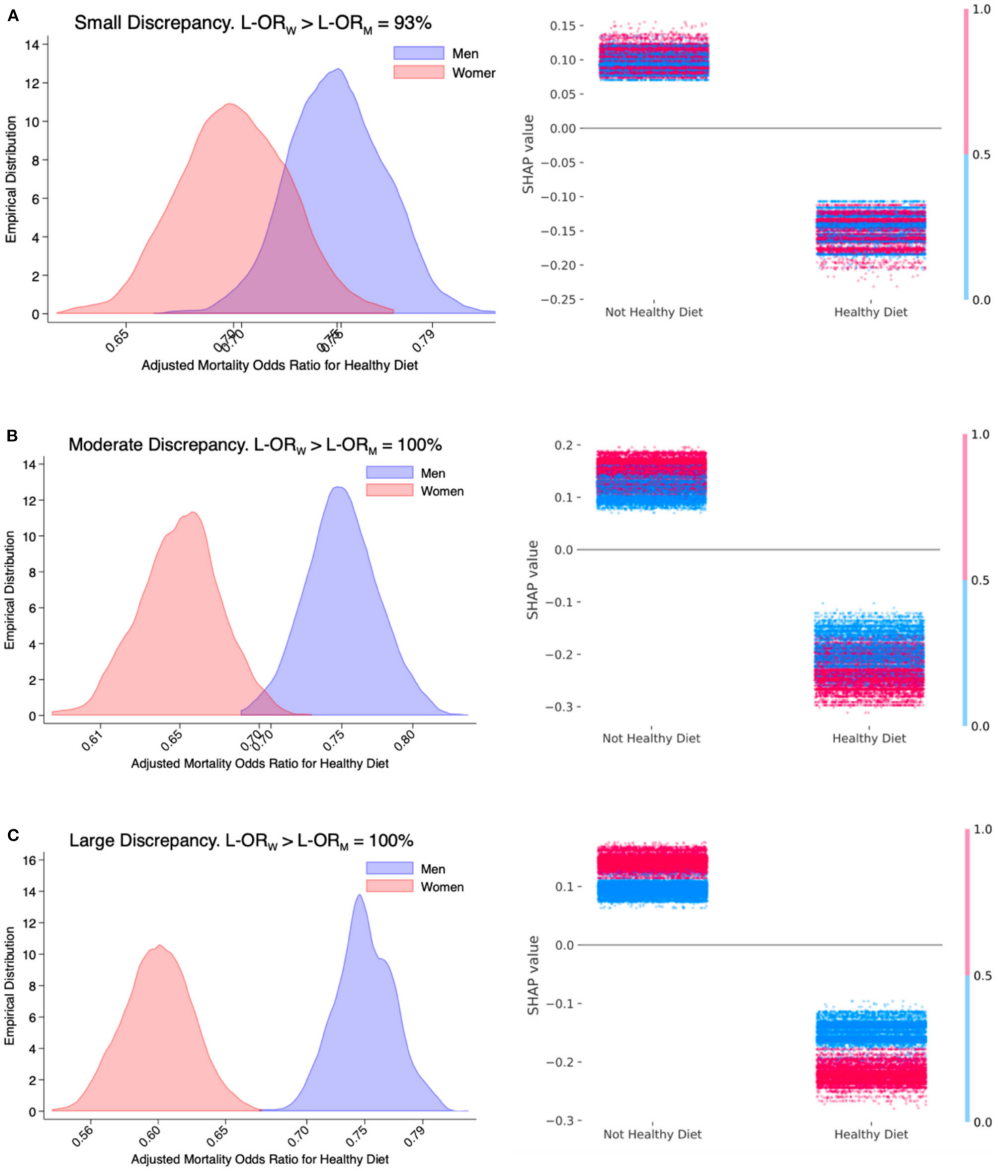
Simulation study—small (**A**; Power = 28%), moderate (**B**; Power = 83%), and large (**C**; Power = 100%) discrepancy by sex in the adjusted mortality odds ratio for healthy diet estimated with a logistic regression model. The left column shows the sampling distribution of the adjusted mortality odds ratio (on the log-scale) conferred by a healthy diet among men and women based on 1,000 simulated studies. The three numbers shown on the x-axis log scale are the 2.5th, 50th, and 97.5th percentiles of the simulated sampling distribution based on a logistic regression model. The percentage indicated in the title is the fraction of studies in which the estimated adjusted mortality odds ratio among women (L-OR_W_) is greater than men (L-OR_M_). The right column shows individual SHAP values derived from extreme gradient boosting on one random sample drawn from the interaction mechanisms that are presented on the left column.

The results of 1,000 Monte-Carlo simulated studies according to low, moderate, and large discrepancy in the effect of healthy diet on mortality risk by female sex, while adjusting for all other relevant factors, are shown in Figure 1.

Based on estimates obtained with a multivariable logistic regression model, the sensitivities of a small, moderate, and large discrepancy were 28, 83, and 100%, respectively. Graphically, this phenomenon is indicated by an increasing separation in the frequencies of estimated adjusted mortality odds ratios conferred by a healthy diet among men and among women. The sensitivity of a positive discrepancy in adjusted mortality odds ratios for healthy diet comparing women vs. men in scenarios of small, moderate, and large interaction effects were 93, 100, and 100%, respectively.

The small interaction effect underlying Figure 1A is 25 and 30% lower adjusted mortality odds ratio conferred by a healthy diet among men and women, respectively. The percentage of sample realizations where the Wald-type test is correctly rejecting the hypothesis of no interaction effect was 28%. In 93% of the simulated studies, the estimated adjusted mortality odds ratio conferred by a healthy diet was correctly greater among women than men. Regarding the large interaction effect underlying Figure 1C, the sensitivity of a such large discrepancy as well as the sensitivity of a positive discrepancy were both 100%.

The second column of Figure 1 shows the individual SHAP values estimated based on XGBoost on a random sample drawn from the 3 interaction mechanisms described above. The fact that the distributions of SHAP values conferred by a healthy diet (right cloud) are consistently lower than the SHAP values conferred by a not-healthy diet (left cloud) indicates an adjusted inverse association of a healthy diet with mortality risk. The increasing magnitude of the interaction effect when moving from Figures 1A–C can be visually appreciated by the increasing vertical distinction in blue (men)/red (women) colors. The stronger protective effect of a healthy diet in women is indicated by the greater distance between the red points among those with and without a healthy diet. This is better appreciated in Figure 1C where the distance between red points (women) is consistently greater than the distance between blue points (men).

**Figure 2:**
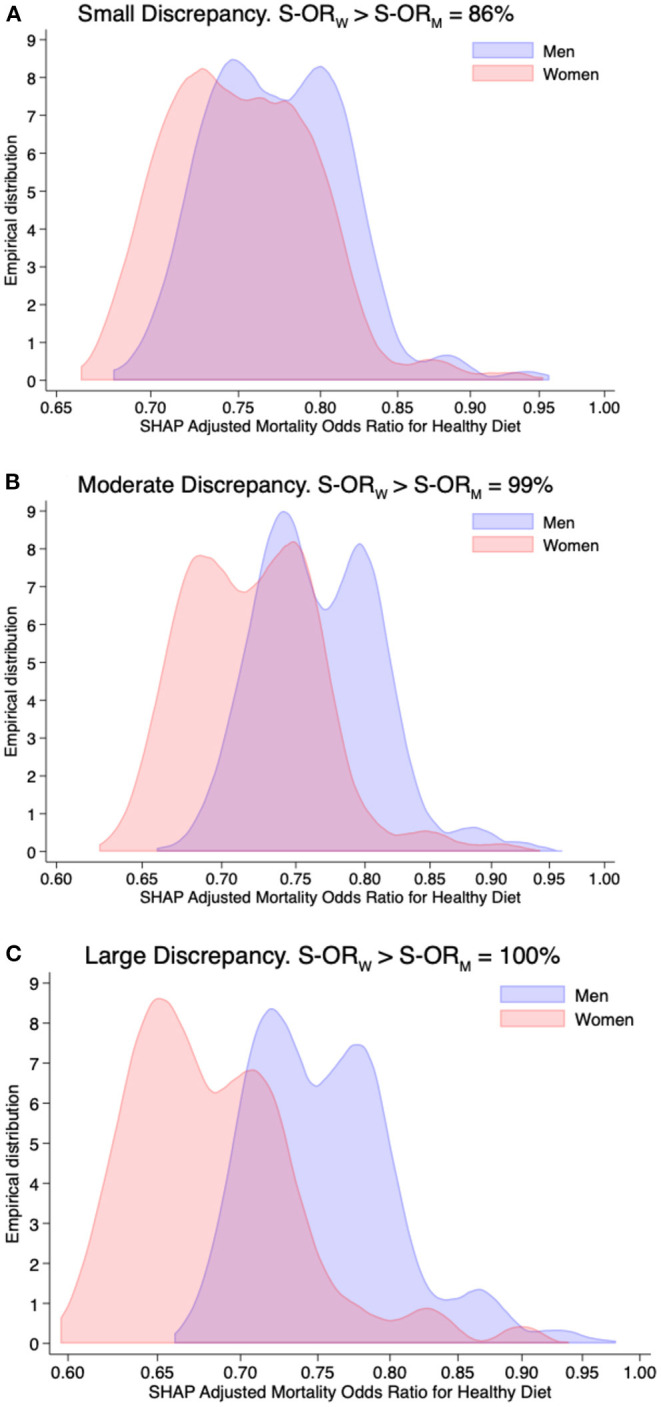
Simulation study—small **(A)**, moderate **(B)**, and large **(C)** discrepancies by sex in the adjusted mortality adjusted odds ratio (on the log-scale) for healthy diet computed in 1,000 studies, each of size of 47,770 individuals and with SHAP values derived from extreme gradient boosting. The x-axis is on the log scale. The percentage indicated in the title is the fraction of times in which the SHAP-based adjusted mortality odds ratio among women (S-OR_W_) is greater than men (S-OR_M_).

Figure 2 provides the simulated sampling distribution of the SHAP-based adjusted mortality odds ratios conferred by a healthy diet among men and women. The sensitivity of a positive discrepancy in small, moderate, and large interaction effects were 86, 99, and 100%, respectively. In contrast to Figure 1, the sampling distributions of SHAP-based adjusted mortality odds ratios conferred by a healthy diet are far from being approximated (on a log scale) by a normal distribution, and the magnitude of the interaction effect, as separation in central tendency, tends to be lower.

### Performance of the Simulation Algorithms

In terms of execution speed, to conduct one simulation study (MacBook Pro 2019, 2.6 GHz 6-Core Intel Core i7) using logistic regression model took about 0.15 s, whereas using XGBoost took about 13 s. Conducting 1,000 simulations required between 3.5 and 4 h for each scenario. In the 1,000 simulated studies using the logistic regression model the bias—defined as the average distance between each simulated interaction effect relative to its true value—was 0.004, −0.002, and 0.001 for the three scenarios of low, moderate, and large discrepancies, respectively. It was difficult to assess bias about the interaction effect derived from XGBoost simply because data were generated according to a conditional probabilistic model, while the SHAP-based odds ratio are marginal effects. The algorithm converged in all the simulated studies and scenarios. The code written in Python is available in the Supplementary Material.

### Empirical Study

Among men, the estimated adjusted mortality odds conferred by a healthy diet was 25% lower (L-OR_M_ = 0.75, 95% CI = 0.70, 0.80). Among women, the estimated adjusted mortality odds conferred by healthy diet was 30% lower (L-OR_W_ = 0.70; 95% CI = 0.65, 0.75). The result of the Wald-type test indicates a compatibility between this sample of data and the hypothesis of no interaction effects between sex and healthy diet in predicting mortality risk upon adjustment for all the relevant factors (*z* = −1.42, *p*-value = 0.156). The *p*-value larger than the nominal 0.05, however, should not be taken, in itself, as a strong indication of the absence of interaction because the ability to detect an adjusted discrepancy of this magnitude (Figure 1A) has been shown to be quite low in the corresponding simulation study. A table of estimates of the estimated multivariable logistic regression model (Area Under Curve = 0.80) is presented in the Supplementary Material.

**Figure 3:**
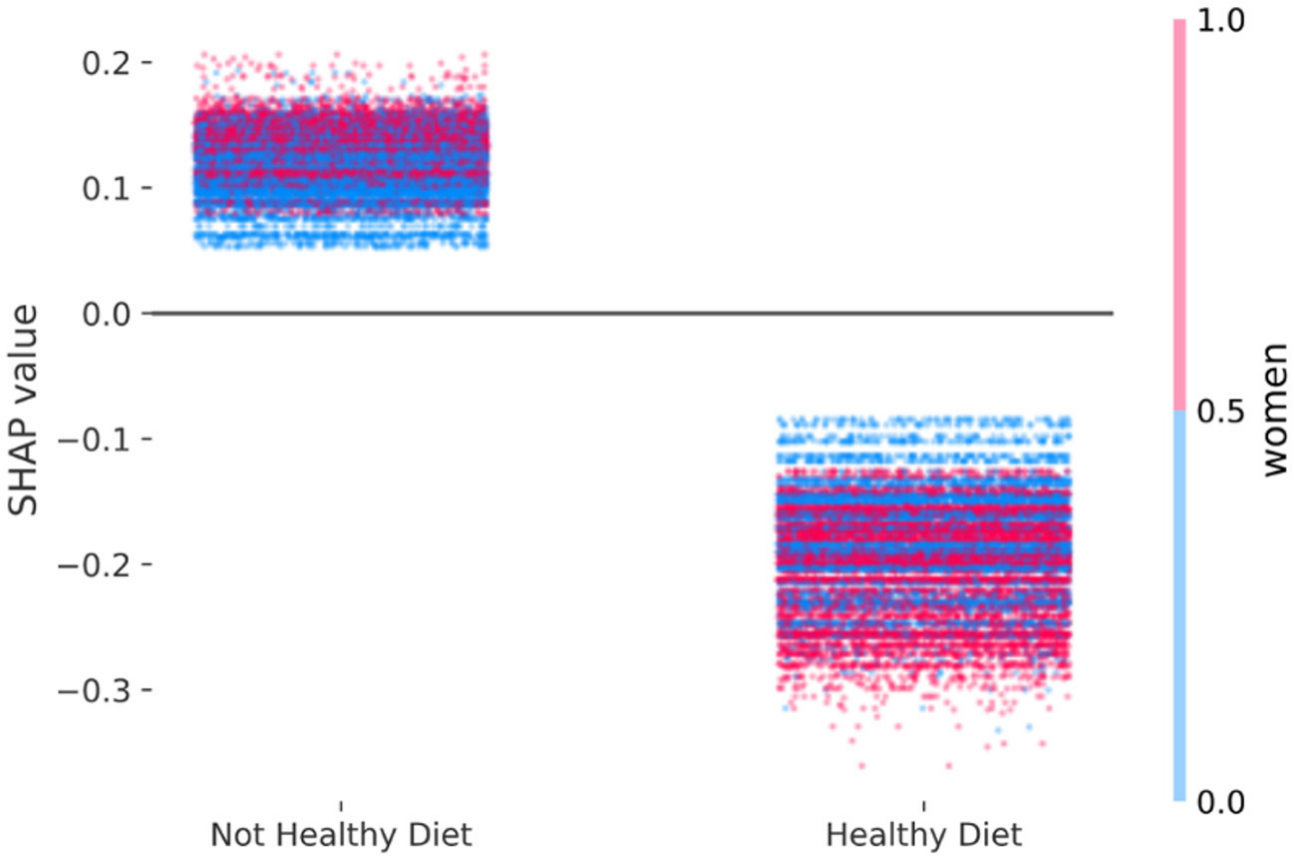
Empirical study—multivariable adjusted association of a healthy diet with mortality risk according to female sex estimated with the SHAP values derived from extreme gradient boosting based on 47,770 participants of the population-based cohorts SMC and COSM. The computed SHAP-based adjusted mortality odds ratio among women (S-OR_W_ = 0.72) is greater than men (S-OR_M_ = 0.74).

Figure 3 shows the adjusted beneficial effect of a healthy diet on mortality risk based on the SHAP values computed after one run of XGBoost on the empirical data (Area Under Curve = 0.74). This indication emerged by the fact that the cloud of SHAP values among those with a healthy diet are consistently lower, meaning lower mortality, than the SHAP values among those with a not healthy diet. Since one may distinguish a cluster of blue dots (men) at the bottom of the not healthy diet (left cloud) and a cluster of red dots (women) at the bottom of the healthy diet (right cloud), Figure 3 suggests a slightly stronger adjusted protective effect of healthy diet on mortality risk among women, in comparison to men. The derived SHAP-based adjusted mortality odds ratio among women (S-OR_W_ = 0.72) was slightly greater than men (S-OR_M_ = 0.74). An attempt to locate the empirical SHAP values shown in Figure 3 into simulated scenarios shown on the left column of Figure 1 suggests that the sensitivity to discern an interaction effect of a similar size to the one observed was low, but the sensitivity of a positive discrepancy was high.

## Discussion

The ability of SHAP methods based on extreme gradient boosting to indicate the presence of an interaction effect of a certain size was proportional to the sensitivity or traditional power of a statistical test in a logistic regression model. Conversely, the ability of SHAP methods to correctly identify the sign or direction of an interaction was very high in all the scenarios, characterized by either small or large interaction effects. The results obtained with the empirical data appeared to be in line with a small interaction effect and a slightly stronger inverse adjusted association between healthy diet and mortality risk among women.

The strength of this study was the simulation of a complex interaction mechanism grounded in the specific characteristics of a real, large population-based prospective epidemiological study. The simulation study allowed us to appreciate the ability of a statistical model or a machine learning algorithm to pinpoint the true data generating mechanism underlying the outcome observations. The stronger the magnitude of the interaction effect, the easier it was for the chosen method, when applied to a particular sample, to correctly indicate the presence of such interaction effect. Since sample size corresponding to the actual analytical sample was fixed, the sensitivity to detect an interaction effect increased with its size.

Consistency between insights provided by visualizations of SHAP values and prior literature is often highlighted in support of the application of machine learning methods. By deriving and describing a numerical summary measure, such as an adjusted mortality odds ratio, we were able to complement the graphical intuitions provided by increasingly popular dependence plots of individual SHAP values (3, 17). The sensitivity of correctly identifying the sign or direction of an interaction effect was very high in all scenarios using either a logistic regression model or XGBoost. It should be emphasized that the interaction term (between healthy diet and sex) was explicitly specified when defining the logistic regression model, whereas the XGBoost was trained without any interaction term. Therefore, the very good performance of the logistic regression model was not surprising, since the model was specified in perfect agreement with the data generating mechanism underlying the outcome observations. In other words, a logistic regression model without including the right interaction term would not be able to uncover what the combination of SHAP and XGBoost uncovered without including any prior knowledge.

The adjusted mortality odds ratios estimated with a logistic regression model and derived from SHAP-values were numerically similar and pointed in the same direction. It should be noted, however, that they are conceptually and mathematically different. A logistic regression model is parametrized directly in terms of the parameter of interest. SHAP values reflect the relative importance of each predictor through its marginal contribution.

Our focused simulation study, in line with the characteristics of a real epidemiological study, provided a reasonable background to carefully interpret visualizations of SHAP values based on machine learning methods computed from the data at hand. Recognizing the difficulty in discerning a genuine interaction effect of a certain size of substantial importance can help the investigator to avoid binary claims (presence/absence of interaction) about plausible, yet unknown, mechanisms underlying the data.

A limitation of our simulation study was that the distribution of all the predictors were dichotomized to simplify the analysis and coding. There is no doubt that different categorizations of the predictors or modeling them as quantitative values, possibly considering non-linearities, would have led to different estimates in our empirical study. Since our goal was to conduct a simulation study focusing on the interaction effect between healthy diet and sex, dichotomization of predictors greatly simplified its implementation and analysis. Our simplified model, however, presented a relatively high ability to discriminate the mortality outcomes. Another limitation was that hyperparameters of XGBoost were obtained by means of two-fold cross validation, which might be suboptimal and explain why the area under the curve from XGBoost was, at least in the empirical data, slightly lower than that from logistic regression. However, performing predictions was not a primary concern in our simulation study.

In conclusion, in this realistic simulation study we found that the ability of the SHAP values to detect an interaction effect was proportional to its magnitude. In contrast, the ability to identify the sign or direction of such interaction effect was very high in all the simulated scenarios. The results obtained with the empirical data appeared to be in line with a small interaction effect.

## Data Availability Statement

The data analyzed in this study is subject to the following licenses/restrictions: original individual data that inspired the simulation study cannot be shared. Algorithm to simulate individual data is available in the Supplementary Material. Requests to access these datasets should be directed to alicja.wolk@ki.se.

## Author Contributions

NO, AM, and AW defined the question, conceptualize the simulation study, and drafted the manuscript. NO and AM wrote the code to simulate and analyze a realistic large prospective study.

## Funding

This study was supported by grants from the Swedish Research Council (grant no 2017-06100). We acknowledge the national research infrastructure SIMPLER (www.simpler4health.se) for providing data. SIMPLER receives funding from the Swedish Research Council (grant no 2017-00644).

## Conflict of Interest

AM was employed by Managed Self Ltd T/A Klarity, Bournemouth, UK. The remaining authors declare that the research was conducted in the absence of any commercial or financial relationships that could be construed as a potential conflict of interest.

## Publisher's Note

All claims expressed in this article are solely those of the authors and do not necessarily represent those of their affiliated organizations, or those of the publisher, the editors and the reviewers. Any product that may be evaluated in this article, or claim that may be made by its manufacturer, is not guaranteed or endorsed by the publisher.
