# Supplementary material for: Interaction Analysis Based on Shapley Values and Extreme Gradient Boosting: A Realistic Simulation and Application to a Large Epidemiological Prospective Study
Source: Front Nutr. 2022 Jul 18;9:871768. doi: 10.3389/fnut.2022.871768 (PMC9340268; doi:10.3389/fnut.2022.871768)
Supplement: Supplementary file 1 [file Data_Sheet_1.docx]

**Supplementary Material**

**Description of the epidemiological data**

This study included participants from two large population-based cohorts of Swedish men and women, the Cohort of Swedish Men (COSM) and the Swedish Mammography Cohort (SMC). The analytical sample was based on 47,770 participants and frequency distributions of data are shown below.

| **Variables** | **Count (%)** |
| --- | --- |
| Mortality status (dead vs alive) | 14,497 (30) |
| Female sex (women vs men) | 23,045 (48) |
| Age (< 65 vs $\geq$ 65 years) | 32,583 (68) |
| No small body mass index (BMI $\geq$ 20 kg/m^2^) | 45,831 (96) |
| Small waist circumference (<88 cm for women and <102 cm for men) | 35,257 (74) |
| Educational level (high school/university vs primary) | 14,888 (31) |
| Leisure walking ($\geq$ 20 min/d vs never or <20min/d) | 42,656 (89) |
| Never smoking (never vs former/current) | 22,018 (46) |
| Any alcohol drinking (moderate vs either moderate-to-never or moderate-to-heavy) | 16,822 (35) |
| Sleep duration (7 hours vs either <7 or >7 hours) | 19,382 (41) |
| Living with someone (yes vs no) | 37,680 (79) |
| Healthy diet (top quartile of recommended food items and bottom quartile of non-recommended food items vs other quartile). | 20,080 (42) |

**Table**. Adjusted Mortality Odds Ratios (95% confidence interval and test of the null hypothesis) estimated with a multivariable logistic regression model based on 47,770 middle-aged and elderly people from the COSM and SMC cohorts. The Area Under the Curve was 0.80.

|  | **Odds Ratio** | **Std. err.** | **z** | **p-value** | **95% Conf.** | **Interval** |
| --- | --- | --- | --- | --- | --- | --- |
| age | 0.104 | 0.003 | -92.18 | <0.001 | 0.099 | 0.109 |
| nosmallbmi | 0.614 | 0.036 | -8.38 | <0.001 | 0.548 | 0.688 |
| smallwaist | 0.803 | 0.021 | -8.28 | <0.001 | 0.762 | 0.846 |
| educated | 0.696 | 0.019 | -13.09 | <0.001 | 0.660 | 0.735 |
| walk | 0.702 | 0.026 | -9.74 | <0.001 | 0.653 | 0.754 |
| neversmoke | 0.745 | 0.018 | -12 | <0.001 | 0.710 | 0.782 |
| drink | 0.853 | 0.022 | -6.11 | <0.001 | 0.810 | 0.897 |
| sleep7h | 0.866 | 0.021 | -5.94 | <0.001 | 0.825 | 0.908 |
| livesomeone | 0.653 | 0.018 | -15.26 | <0.001 | 0.618 | 0.690 |
| women | 0.626 | 0.020 | -14.83 | <0.001 | 0.588 | 0.666 |
| healthydiet | 0.748 | 0.026 | -8.49 | <0.001 | 0.699 | 0.800 |
| women_healthydiet | 0.933 | 0.046 | -1.42 | 0.156 | 0.847 | 1.027 |
| Intercept | 12.061 | 0.935 | 32.13 | <0.001 | 10.362 | 14.039 |
|  |  |  |  |  |  |  |

**Description of the simulation study**

The sample size is fixed at 47,770 individuals. The following 11 baseline binary predictors are randomly generated from a Bernoulli variable according to the frequency distributions shown above for the epidemiological studies.

women ~ Bernoulli(0.48)

age ~ Bernoulli(0.68)

nosmallbmi ~ Bernoulli(.96)

smallwaist ~ Bernoulli(0.74)

educated ~ Bernoulli(0.31)

walk ~ Bernoulli(0.68)

neversmoke ~ Bernoulli(0.45)

drink ~ Bernoulli(0.34)

sleep7h ~ Bernoulli(0.41)

livesomeone ~ Bernoulli(0.79)

healthydiet ~ Bernoulli(0.42)

The binary outcome of mortality is also randomly generated from a Bernoulli variable with a probability *p* according to the following data generating mechanism

death ~ Bernoulli(*p*) and *p* = $e^{x\beta}/(1+e^{x\beta})$

with $x\beta$, linear predictor of the logit (log odds) of the outcome probability is varied according to the following three scenarios characterized by a progressively stronger inverse association between healthy diet and mortality among female sex. The values of the regression coefficients, defining strength and direction of relationships, are based on estimates obtained from the epidemiological data. The adjusted mortality odds ratio comparing healthy diet vs non-healthy diet among men and women are compactly denoted as OR_M_ and OR_W_, respectively.

1. **Small discrepancy**. OR_M_ = $e^{-0.29}=0.75$ and OR_W_ = $e^{-0.29-0.07}=0.70$

$$x\beta=2.28-2.27\left( \mathrm{age} \right)-0.49\left( \mathrm{nosmallbmi} \right)-0.23\left( \mathrm{smallwaist} \right)-0.36\left( \mathrm{educated} \right)-0.12\left( \mathrm{walk} \right)-0.30\left( \mathrm{neversmoke} \right)-0.17\left( \mathrm{drink} \right)-0.15\left( sleep7h \right)-0.43\left( \mathrm{livesomeone} \right)-0.47\left( \mathrm{women} \right)-0.29\left( \mathrm{healthydiet} \right)-0.07\left( \mathrm{women} \right)\left( \mathrm{healthydiet} \right)$$

1. **Moderate discrepancy.** OR_M_ = $e^{-0.29}=0.75$ and OR_W_ = $e^{-0.29-0.14}=0.65$

$$x\beta=2.28-2.27\left( \mathrm{age} \right)-0.49\left( \mathrm{nosmallbmi} \right)-0.23\left( \mathrm{smallwaist} \right)-0.36\left( \mathrm{educated} \right)-0.12\left( \mathrm{walk} \right)-0.30\left( \mathrm{neversmoke} \right)-0.17\left( \mathrm{drink} \right)-0.15\left( sleep7h \right)-0.43\left( \mathrm{livesomeone} \right)-0.47\left( \mathrm{women} \right)-0.29\left( \mathrm{healthydiet} \right)-0.14\left( \mathrm{women} \right)\left( \mathrm{healthydiet} \right)$$

1. **Large discrepancy.** OR_M_ = $e^{-0.29}=0.75$ and OR_W_ = $e^{-0.29-0.22}=0.60$

$$x\beta=2.28-2.27\left( \mathrm{age} \right)-0.49\left( \mathrm{nosmallbmi} \right)-0.23\left( \mathrm{smallwaist} \right)-0.36\left( \mathrm{educated} \right)-0.12\left( \mathrm{walk} \right)-0.30\left( \mathrm{neversmoke} \right)-0.17\left( \mathrm{drink} \right)-0.15\left( sleep7h \right)-0.43\left( \mathrm{livesomeone} \right)-0.47\left( \mathrm{women} \right)-0.29\left( \mathrm{healthydiet} \right)-0.22\left( \mathrm{women} \right)\left( \mathrm{healthydiet} \right)$$

**Python code**

import pandas as pd

import numpy as np

import os

import matplotlib.pyplot as plt

import numpy as np

import statsmodels.api as sm

import statsmodels.formula.api as smf

from scipy.stats import norm

import sklearn

from sklearn import metrics

from sklearn.linear_model import LogisticRegression

from sklearn.model_selection import train_test_split, GridSearchCV

import xgboost as xgb

import shap

def invlogit(xb):

return np.exp(xb) / (1 + np.exp(xb))

def sim_dgm():

n = 47770

one = np.ones(n)

women = np.random.binomial(1, .48, n)

age = np.random.binomial(1, .68, n)

nosmallbmi = np.random.binomial(1, .96, n)

smallwaist = np.random.binomial(1, .74, n)

educated = np.random.binomial(1, .31, n)

walk = np.random.binomial(1, .68, n)

neversmoke = np.random.binomial(1, .45, n)

drink = np.random.binomial(1, .34, n)

sleep7h = np.random.binomial(1, .41, n)

livesomeone = np.random.binomial(1, .79, n)

healthydiet = np.random.binomial(1, .42, n)

inter = women*healthydiet # interaction effect

# a) Small discrepancy

xb = 2.28-2.27*age-0.49*nosmallbmi -.23*smallwaist -.36*educated-0.12*walk-0.30*neversmoke -0.17*drink -.15*sleep7h -0.43*livesomeone-0.47*women -0.29*healthydiet -0.07*inter

# b) Moderate discrepancy

# xb = 2.28-2.27*age-0.49*nosmallbmi -.23*smallwaist -.36*educated-0.12*walk-0.30*neversmoke -0.17*drink -.15*sleep7h -0.43*livesomeone-0.47*women -0.29*healthydiet -0.14*inter

# c) Large discrepancy

# xb = 2.28-2.27*age-0.49*nosmallbmi -.23*smallwaist -.36*educated-0.12*walk-0.30*neversmoke -0.17*drink -.15*sleep7h -0.43*livesomeone-0.47*women -0.29*healthydiet -0.22*inter

y = np.random.binomial(1, invlogit(xb), n)

columns = ['cons', 'age', 'nosmallbmi', 'smallwaist', 'educated', 'walk', 'neversmoke', 'drink', 'sleep7h', 'livesomeone', 'women', 'healthydiet', 'inter']

list_pred = (one, age, nosmallbmi, smallwaist, educated, walk, neversmoke, drink, sleep7h, livesomeone, women, healthydiet, inter)

X = pd.DataFrame(np.column_stack(list_pred), columns = columns )

df = pd.DataFrame({

"age": age,

"women": women,

"nosmallbmi": nosmallbmi,

"smallwaist": smallwaist,

"educated": educated,

"walk": walk,

"neversmoke": neversmoke,

"drink": drink,

"sleep7h": sleep7h,

"livesomeone": livesomeone,

"healthydiet": healthydiet,

"early_death": y

})

# Logistic regression

model_lr = sm.Logit(y, X).fit()

print(model_lr.summary())

b = model_lr.params

l_lnor_hd_men = b[11]

l_lnor_hd_women = b[11] + b[12]

# XGBOOST

train, test = train_test_split(df, test_size=0.2)

model = xgb.XGBClassifier()

xgb_grid = GridSearchCV(

model,

{

"max_depth": [3, 6],

"n_estimators": [50, 100, 250],

"learning_rate": [0.01, 0.05],

}

,

cv=2, n_jobs=-1, verbose=True

)

xgb_grid.fit(train.drop("early_death", axis=1), train["early_death"])

model = xgb.XGBClassifier(**xgb_grid.best_params_)

model.fit(train.drop("early_death", axis=1), train["early_death"])

X = train.drop("early_death", axis=1)

for c in X.columns:

X[c]=X[c].astype(float)

explainer = shap.TreeExplainer(model)

shap_values = explainer.shap_values(X)

X.reset_index(drop=True, inplace=True)

hd_women_rows = X.index[(X.healthydiet==1)&(X.women==1)].tolist()

hd_men_rows = X.index[(X.healthydiet==1)&(X.women==0)].tolist()

nhd_women_rows = X.index[(X.healthydiet==0)&(X.women==1)].tolist()

nhd_men_rows = X.index[(X.healthydiet==0)&(X.women==0)].tolist()

s_lnor_hd_women = np.mean(shap_values[hd_women_rows,10]) - np.mean(shap_values[nhd_women_rows,10])

s_lnor_hd_men = np.mean(shap_values[hd_men_rows,10]) - np.mean(shap_values[nhd_men_rows,10])

return(l_lnor_hd_men, l_lnor_hd_women, s_lnor_hd_men , s_lnor_hd_women)

# Loop to simulate 1,000 times

nsim = 1000

b = []

for i in range(nsim):

b = np.append(b, np.array(sim_dgm()), axis=0)
